# Contrast-enhanced ultrasound combined targeted microbubbles for diagnosis of highly aggressive papillary thyroid carcinoma

**DOI:** 10.3389/fendo.2023.1052862

**Published:** 2023-03-03

**Authors:** Jiaojiao Ma, Yuan Wang, Xuehua Xi, Jiajia Tang, Linping Wang, Liangkai Wang, Di Wang, Xiaolong Liang, Bo Zhang

**Affiliations:** ^1^ Department of Ultrasound, China-Japan Friendship Hospital, Beijing, China; ^2^ National Center for Respiratory Medicine, National Clinical Research Center for Respiratory Diseases, Institute of Respiratory Medicine of Chinese Academy of Medical Sciences, Beijing, China; ^3^ Department of Ultrasound, Peking University Third Hospital, Beijing, China; ^4^ Chinese Academy of Medical Sciences & Peking Union Medical College, Beijing, China; ^5^ Department of Ultrasound, First Affiliated Hospital of Xiamen University, Xiamen, China

**Keywords:** papillary thyroid carcinoma, highly aggressive, contrast-enhanced ultrasound, galectin-3, targeted microbubble

## Abstract

**Background:**

Accurate diagnosis of highly aggressive papillary thyroid cancer (PTC) may greatly help avoid overdiagnosis and overtreatment of PTC. However, there is still a lack of a convenient and accurate method. Targeted microbubbles, an emerging ultrasound contrast agent, have the potential to accurately diagnose highly aggressive PTC.

**Purpose:**

To design and prepare a targeted microbubble for specific contrast-enhanced ultrasound (CEUS) imaging of highly invasive PTC.

**Methods:**

Using β-galactoside-binding protein galectin-3 (Gal-3) overexpressed on the surface of highly invasive PTC cells as a target, C12 polypeptide (ANTPCGPYTHDCPVKR) with high affinity and specificity for Gal-3 was coupled to the surface of lipid microbubbles to prepare targeted microbubbles (Gal-3-C12@lipo MBs). The targeted microbubbles were prepared by thin-film hydration method and mechanical shaking method. The morphology, diameter, concentration and stability of microbubbles were investigated by fluorescence microscopy and an AccuSizer. The biosafety of microbubbles was studied using BCPAP cells through CCK8 assay. Confocal laser scanning microscope and flow cytometry were applied to research the cellular uptake of microbubbles to investigate the targeting ability to highly aggressive PTC. Finally, the specific contrast-enhanced ultrasound imaging of microbubbles in highly invasive PTC was validated on the mice bearing subcutaneous BCPAP tumor model *via* a clinically ultrasound imaging system.

**Results:**

Gal-3-C12@lipo MBs were successfully prepared which showed a well-defined spherical morphology with an average diameter of 1.598 ± 0.848 μm. Gal-3-C12@lipo MBs showed good stability without rupture within 4 hours after preparation. At the cellular level, Gal-3-C12@lipo MBs exhibited favorable biosafety and superior targeting ability to BCPAP cells, with 2.8-fold higher cellular uptake than non-targeted lipid microbubbles (Lipo MBs). At the animal level, Gal-3-C12@lipo MBs significantly improved the quality of contrast-enhanced ultrasound imaging in highly invasive PTC, with an echo intensity of tumor significantly higher than that of Lipo MBs.

**Conclusion:**

We designed and fabricated a novel targeted microbubble for the specific ultrasound imaging diagnosis of highly aggressive PTC. The targeted microbubbles have good stability, superior biosafety and high targeting specificity, which can significantly improve the tumor signal-to-noise ratio of highly invasive PTC, and have the potential to facilitate and accurately diagnose highly invasive PTC.

## Introduction

Thyroid cancer is the most common endocrine cancer worldwide. Over the past 40 years, a significant increase incidence of thyroid cancer has been reported, especially for papillary thyroid cancer (PTC), with a reported growing incidence in men and women since 2005 tripling from 1983 to 2012 (1, 2). It is generally believed that this marked rise in incidence is caused by an increasing detection of small and clinically insignificant thyroid nodules by ultrasound imaging and not all detected nodules need to be treated immediately. It is reported that more than 470,000 women and 90,000 men may have been over-diagnosed over the past two decades, and the overall costs for treating those patients are substantial (3–6). Conventional handling of nonaggressive nodules leads to unnecessary biopsies, wasted healthcare costs, and degradation of patients’ quality of life (7). How to accurately detect invasive PTC is a problem that needs to be solved.

Clinically, ultrasound has been established as a baseline imaging technique for thyroid nodules. Traditional ultrasound is considered as a valuable new approach in the determination of benign *vs*. malignant thyroid nodules. Contrast-enhanced ultrasound (CEUS) is a new technique, which has been used in the differential diagnosis of benign and malignant thyroid nodules (8, 9). The main advantage of CEUS is the ability to assess real-time sequence and intensity of vascular perfusion and hemodynamics in the thyroid nodule. Conventional ultrasound combined with CEUS can improve the accuracy of differential diagnosis of benign and malignant thyroid nodules (10), but still cannot distinguish the invasiveness of PTC.

At present, the methods to identify the invasiveness of PTC include serum biomarker detection, targeted radionuclide imaging and so on, but the results are not satisfactory (11–13). CEUS combined with targeted microbubbles, binding specifically to cell surface receptors *via* targeting peptides or antibodies on the microbubble surface, has been previously demonstrated to be an emerging, potentially highly smart and effective approach for the diagnosis of many kinds of cancers (14, 15), however, their application to identify the invasiveness of PTC was rarely reported.

Galectin 3 (Gal-3), which is highly over-expressed in aggressive PTC but undetectable in normal and benign thyroid conditions, is already clinically adopted as an important diagnostic biomarker to distinguish thyroid malignancy from benign nodules. What’s more, Gal-3 may act as an adhesion molecule in tumor progression and loosen the connection between tumor cells to promote cancer cell metastasis due to its physiological roles (16, 17). Obviously, the Gal-3 level is significantly elevated in cancer tissues and is associated with cancer metastasis (18–20). Therefore, Gal-3 may provide more significant contributions in distinguishing PTC with lymph node metastasis (highly aggressive) or not (less aggressive) (12). Studies have previously shown that radiolabeled antibodies directed against Gal-3 could accumulate in subcutaneous thyroid cancer xenografts in mice and exhibit high sensitivity in distinguishing thyroid cancer from normal thyroid in PET imaging (11, 21, 22). With this background, a CEUS strategy combined with targeted microbubbles that decorated with Gal-3 targeting molecules, which has not yet been reported, has the potential to detect high invasive PTC.

**Figure 1:**
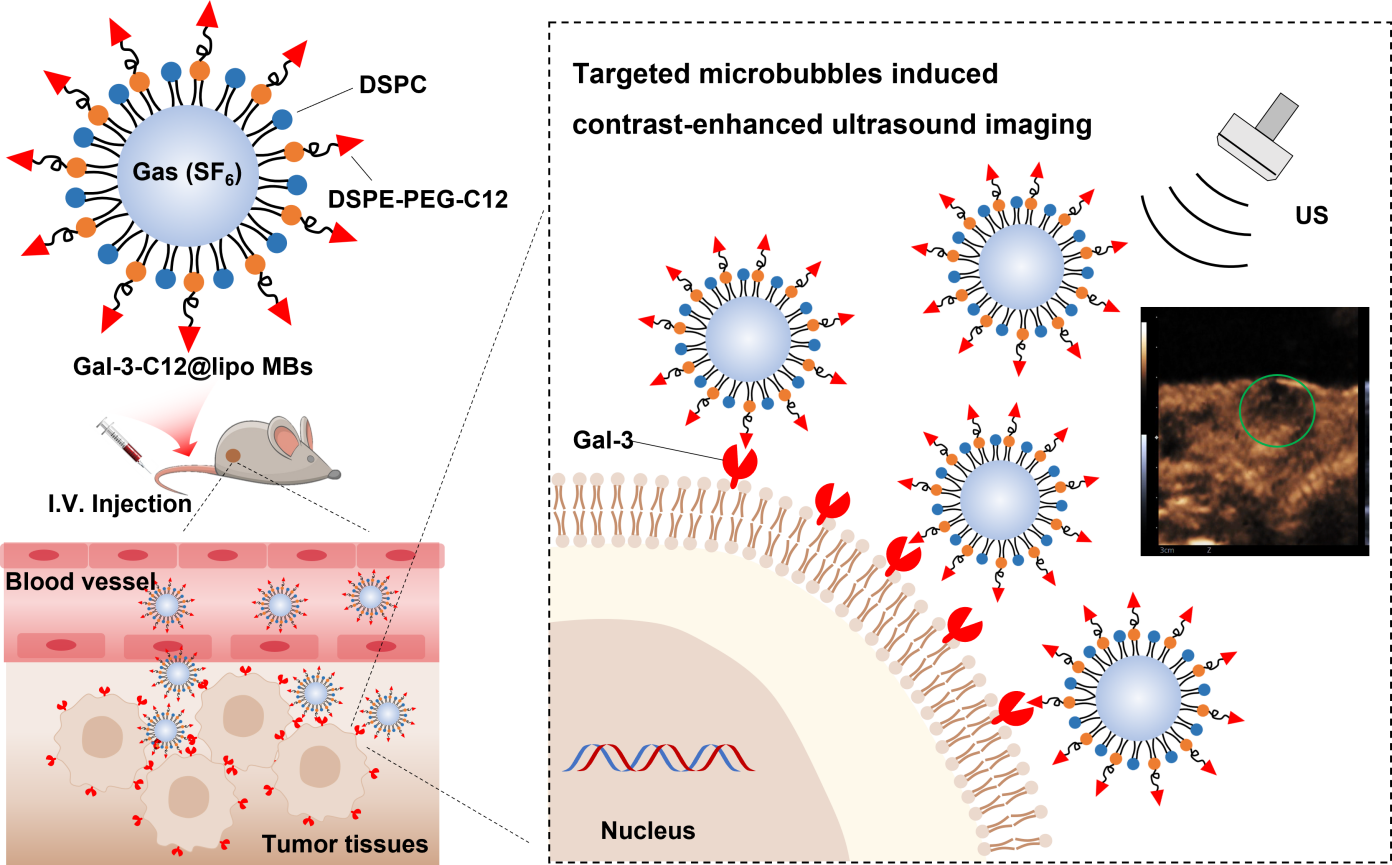
Schematic illustration of the structure of Gal-3-C12@lipo MBs and the targeted microbubbles induced contrast-enhanced ultrasound imaging of highly aggressive papillary thyroid cancers.

The purpose of this study is to demonstrate the specificity of PTC detection by targeted microbubbles decorated with Gal-3 targeting polypeptide (C12, ANTPCGPYTHDCPVKR) in thyroid cancer models using CEUS imaging, which is quantitatively analyzed with advanced ultrasound quantification software (VueBox^®^; Bracco, Suisse SA, Geneva, Switzerland). We show that our methodology is highly sensitive in distinguishing specifically between normal thyroid tissue and PTC tumor tissues. This innovation, if optimized for clinical use, promises a selective diagnosis method of imaging Gal-3 positive (aggressively malignant) papillary thyroid nodules *in vivo*, allowing a better preoperative selection of the nodule candidate to surgery (**Figure 1**).

## Results

### Synthesis and characterization of Gal-3-C12@lipo MBs

**Figure 2:**
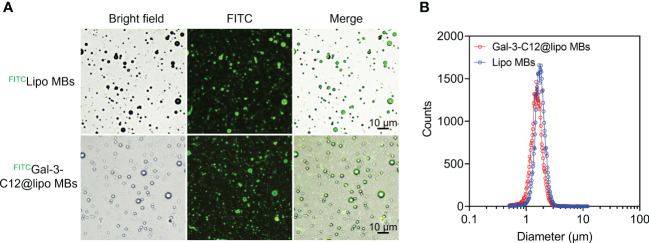
**(A)** Light and fluorescence images of FITC labelled Lipo MBs (^FITC^Lipo MBs) and Gal-3-C12@lipo MBs (^FITC^Gal-3-C12@lipo MBs); **(B)** The diameter distribution of Lipo MBs and Gal-3-C12@lipo MBs measured by an AccuSizer.

Gal-3-C12@lipo MBs were prepared using thin-film hydration method followed by mechanical shaking. To characterize the structure of microbubbles, FITC was used to label DSPE-PEG2000 to prepare FITC labelled Gal-3-C12@lipo MBs (^FITC^Gal-3-C12@lipo MBs). The microscopy images of ^FITC^Gal-3-C12@lipo MBs showed that these microbubbles were dispersive with uniformly spherical morphology, and the green fluorescence from FITC was observed on the shell of the microbubbles, indicating the successfully preparation of Gal-3-C12@lipo MBs (**Figure 2A**). As a non-targeting control, the Lipo MBs without targeted peptide C12 were also uniform-sized microbubbles as exhibited by the microscopy, suggesting the peptide C12 decorated on Gal-3-C12@lipo MBs had no obvious effect on the formation of microbubbles. The mean diameters of Lipo MBs and Gal-3-C12@lipo MBs were 1.777 ± 0.522 μm and 1.598 ± 0.848 μm, respectively, suggesting that the coupling of C12 peptide made Gal-3-C12@lipo MBs smaller in size (**Figure 2B**).

**Figure 3:**
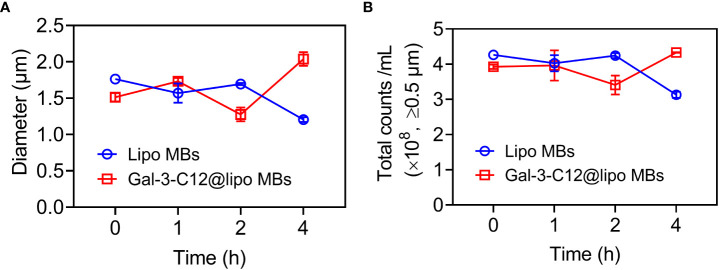
**(A)** The mean diameters change of Lipo MBs and Gal-3-C12@lipo MBs within 4 hours after preparation; **(B)** The microbubble concentrations of Lipo MBs and Gal-3-C12@lipo MBs within 4 hours after preparation.

The stability of microbubbles is essential for enhanced contrast imaging at the target site. Subsequently, the stability of Gal-3-C12@lipo MBs was evaluated by monitoring the mean diameter and concentration of microbubbles. As shown in **Figure 3**, both Lipo MBs and Gal-3-C12@lipo MBs showed slightly size change within 4 hours after preparation. Meanwhile, the concentration of microbubbles with diameters larger than 0.5 μm also displayed insignificant change and maintained at the range of 3.0×10^8^~4.5×10^8^ per mL, further confirming that the Lipo MBs and Gal-3-C12@lipo MBs could keep stable without breaking for at least 4 hours after preparation.

**Figure 4:**
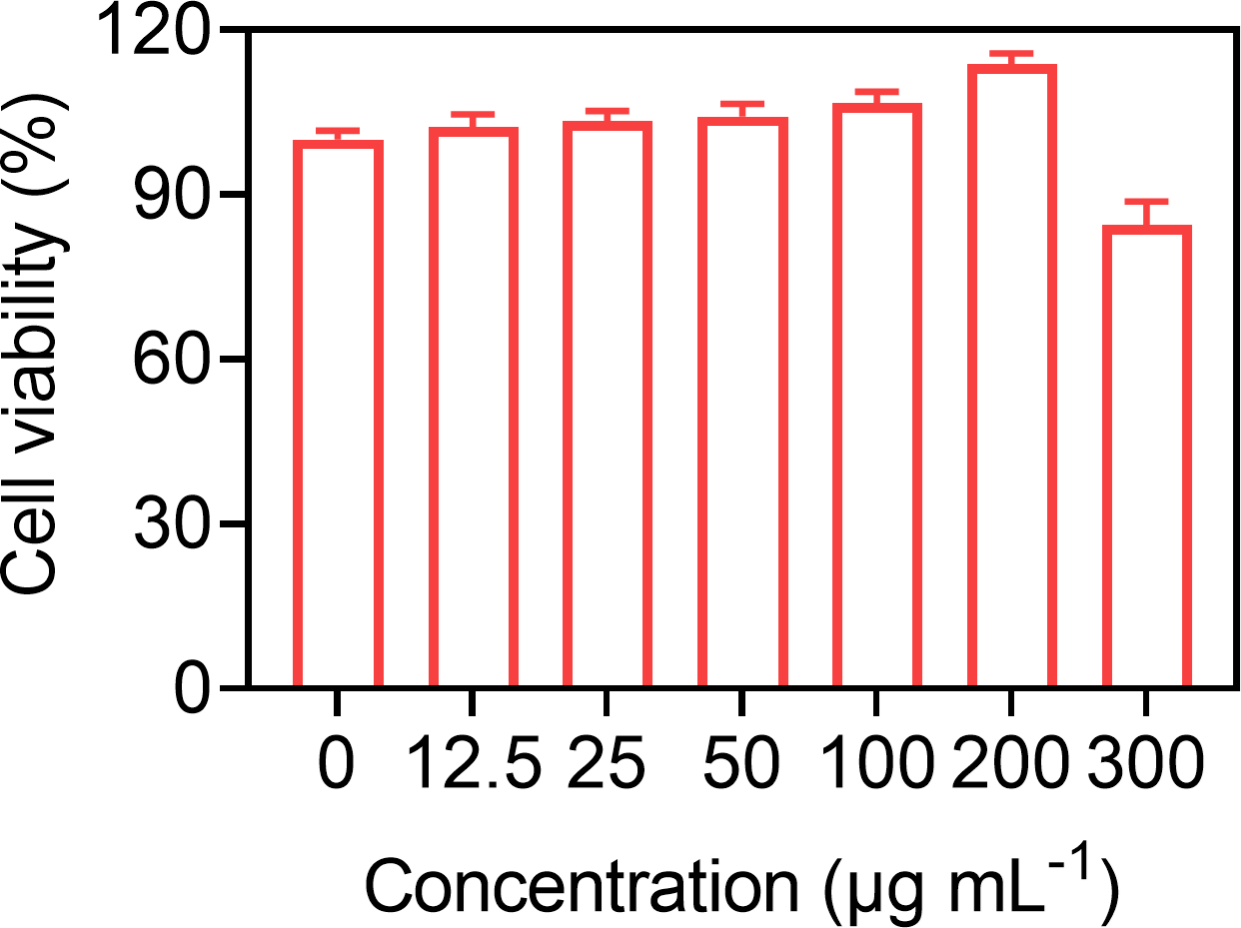
BCPAP cell viability after 24 hours incubation with Gal-3-C12@lipo MBs at different concentrations.

Good biocompatibility is a prerequisite for the clinical translation of microbubbles. So, the biocompatibility of Gal-3-C12@lipo MBs was preliminary demonstrated at the cellular level through incubating various concentrations of Gal-3-C12@lipo MBs with BCPAP cells for 24 h. **Figure 4** showed that Gal-3-C12@lipo MBs caused little damage to cells even at the concentration up to 300 μg mL^-1^, indicating a good biosafety of Gal-3-C12@lipo MBs and great potential for clinical application.

### Targeting ability of Gal-3-C12@lipo MBs in BCPAP cells

**Figure 5:**
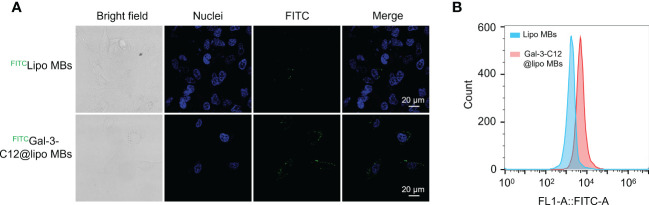
**(A)** Confocal images of BCPAP cells after 6 hours incubation with FITC labelled Lipo MBs and Gal-3-C12@lipo MBs; **(B)** Flow cytometry of BCPAP cells after 6 hours incubation with FITC labelled Lipo MBs and Gal-3-C12@lipo MBs.

To demonstrate the Gal-3 positive PTC tumor cell targeting ability of Gal-3-C12@lipo MBs, CLSM and flow cytometry were applied to conduct cellular uptake experiments using FITC labelled Gal-3-C12@lipo MBs and Lipo MBs. After 6 hours co-incubation, cells which were treated with ^FITC^Gal-3-C12@lipo MBs exhibited remarkably increased green fluorescence intensity compared to ^FITC^Lipo MBs which were not modified with C12 peptide, suggesting that C12 on Gal-3-C12@lipo MBs surface could target to tumor cells and enhance cellular uptake (**Figure 5A**). In addition, the quantitative analysis of fluorescence by flow cytometry also verified this result. The FITC fluorescence intensity of ^FITC^Gal-3-C12@lipo MBs treated group was 2.8 times higher than ^FITC^Lipo MBs treated group (**Figure 5B**). Obviously, the Gal-3-C12@lipo MBs decorated with C12 showed superior targeting capability for highly invasive PTC tumor cells, which was very beneficial for improving the imaging efficiency.

### Gal-3 expression analysis in tumor xenografts

**Figure 6:**
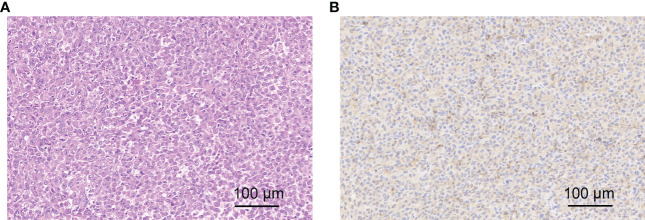
**(A)** H&E staining of BCPAP tumor; **(B)** Gal-3 immunohistochemistry staining of BCPAP tumor.

Galectin-3 (Gal-3) is overexpressed on the surface of highly aggressive PTC tumor cells and can be a potential target for drug diagnosis and therapy. So, H&E staining and immunohistochemistry analysis for Gal-3 were applied to evaluate histopathology of BCPAP tumors. H&E staining image clearly showed the cellular pleomorphism and deep-dyed big nucleolus, which were the typical thyroid cancer phenotype. Besides, immunohistochemistry analysis of tumor sections showed lots of brownish-yellow areas, suggesting the expression of a considerable amount of Gal-3 (**Figures 6A, B**), which would be beneficial for our targeted imaging studies.

### Gal-3-C12@lipo MBs mediated contrast-enhanced ultrasound imaging of PTC

**Figure 7:**
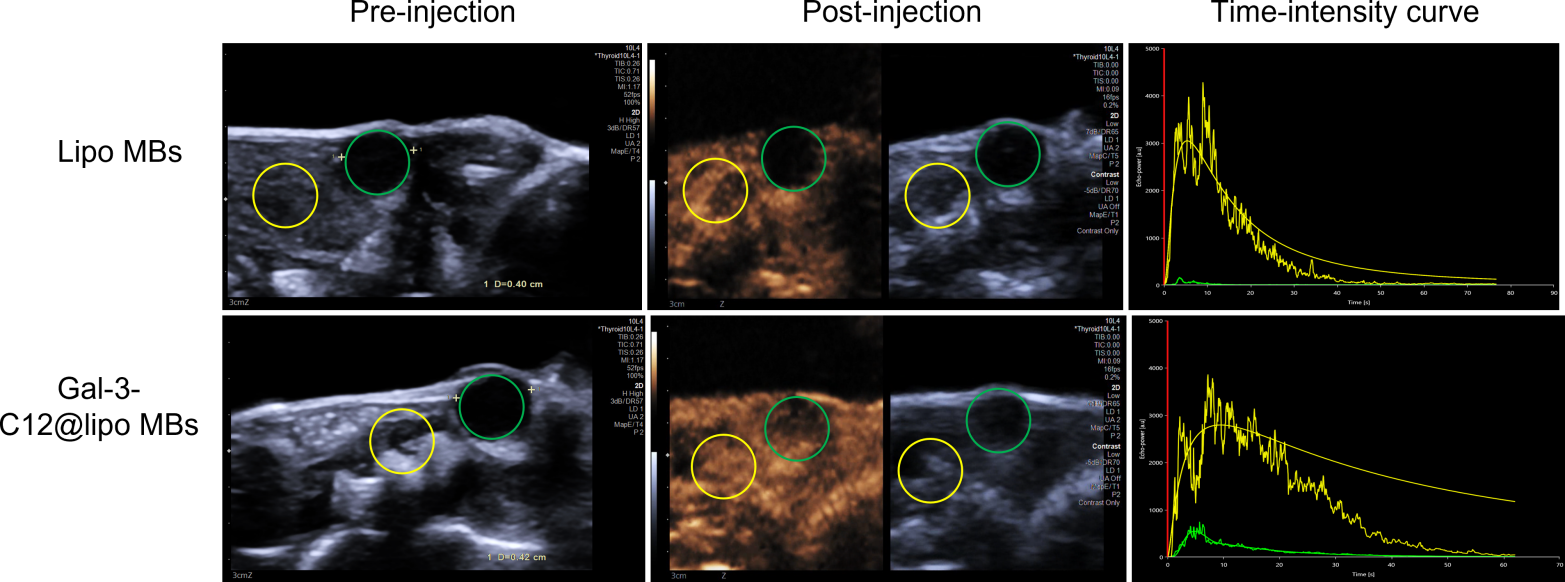
US images in BCPAP subcutaneous tumors captured by a clinical imaging system pre and after i.v. injection of microbubbles about 10 seconds while achieving peak enhancement. The green and yellow circles represent the tumor and normal regions, respectively.

One US system was adopted to investigate the US-targeted contrast-enhanced capability of Gal-3-C12@lipo MBs in the PTC model created in BCPAP subcutaneous tumor mice. Imaging experiments *in vivo* collected images in B-mode and CEUS-mode via a 4-10 MHz linear transducer. Images were analyzed by Vuebox software. As shown in **Figure 7** and **Table 1**, there was significant statistical difference in parameters including mean line, peak enhancement, WiAUC, WiWoAUC, WoAUC, TTP and WiPI between lipo MBs group and Gal-3-C12@lipo MBs group. The mean line and peak enhancement of Gal-3-C12@lipo MBs group were significantly higher than that of lipo MBs, suggesting that more Gal-3-C12@lipo MBs entered the tumor than Lipo MBs; the TTP of Gal-3-C12@lipo MBs group was significantly longer than that of Lipo MBs, and the WiPI of Gal-3-C12@lipo MBs group was also significantly higher than that of Lipo MBs indicating that more Gal-3-C12@lipo MBs continuously entered into the tumor tissues, resulting in longer-lasting imaging effect than Lipo MBs; the WiAUC, WoAUC and WiWoAUC of Gal-3-C12@lipo MBs group were all significantly larger than that of Lipo MBs, further demonstrating that the increased tumor enrichment of Gal-3-C12@lipo MBs could effectively prolong the imaging time window, which could greatly help to distinguish it from both surrounding tissuses and normal thyroid tissues.

**Table 1:** Comparison of CEUS quantitative parameters by Vuebox software in Lipo MBs and Gal-3-C12@lipo MBs.

Parameters	Lipo MBs group	Gal-3-C12@lipo MBs group	*P* value
mean line^*^	0.020(0.156)	0.214(0.347)	0.04
peak enhancement^*^	0.007(0.063)	0.174(0.223)	0.02
WiAUC^*^	0.006(0.197)	0.440(0.716)	0.04
WiWoAUC^*^	0.004(0.302)	0.409(1.325)	0.07
WiR	0.018(0.085)	0.080(0.149)	0.18
WoR	0.005(0.230)	0.079(0.238)	0.18
WoAUC^*^	0.004(0.344)	0.389(1.598)	0.07
rise time	1.149(3.646)	2.008(5.542)	0.31
fall time	1.030(11.022)	2.416(9.339)	0.59
mTTl	1.107(80.255)	0.739(9.361)	0.82
TTP^*^	1.476(2.439)	3.017(6.498)	0.09
WiPI^*^	0.008(0.062)	0.198(0.244)	0.03

*Significantly different between Lipo MBs and Gal-3-C12@lipo MBs group (P<0.01). Data are presented as median (IQR). Lipo MBs, non-targeted liposome microbubbles; Gal-3-C12@lipo MBs, targeted liposome microbubbles carrying C12 (a peptide, ANTPCGPYTHDCPVKR). Peak enhancement (arbitrary units, AU), defined as the maximum intensity of the curve; wash-in area under the curve (WiAUC, AU); wash-out area under the curve (WoAUC, AU); wash-in and wash-out areas under the curve (WiWoAUC, AU); wash-in rate (WiR), in terms of maximum slope (AU); (6) wash-out rate (WoR), in terms of minimum slope (AU); rise time (s); fall time (s); mean transit time local (mTTl, s); time to peak (TTP, s); wash-in perfusion index (WiPI), defined as WiAUC/rise time; and quality of fit (%) between echo-power signal and f (t).

## Discussion

Accurate diagnosis of highly invasive PTC is beneficial to reduce overdiagnosis and overtreatment. At present, a single imaging method is difficult to accurately diagnose the invasiveness of PTC. It has been reported that the combination of magnetic resonance imaging and biomarkers can significantly improve the efficiency of clinical diagnosis of prostate diseases and has been widely studied in the diagnosis of urinary diseases. Therefore, combining different imaging methodologies for the detection of various diseases has become the development trend of clinical diagnosis of diseases. In addition to magnetic resonance imaging, ultrasound imaging is also commonly used in clinical diagnosis of diseases, and ultrasound imaging plays an important role in the diagnosis of thyroid cancer. However, single ultrasound imaging is still insufficient in judging the invasiveness of PTC (23, 24). CEUS combined with targeted microbubbles has been previously demonstrated to be an emerging, potentially highly smart and effective approach for the diagnosis of many kinds of cancers (14, 15), which shows great potential to accurately diagnose highly invasive PTC. For example, Mancini M et al (25) reported the imaging of thyroid tumor angiogenesis with microbubbles targeted to vascular endothelial growth factor receptor type 2 in mice, but did not study the invasiveness of tumors (26). In this study, a targeted microbubble combined with CEUS technique was developed to realize the specific diagnosis of highly invasive PTC.

We selected the highly expressed Gal-3 on the surface of invasive PTC as the target (11, 27), and modified the polypeptide C12 with high affinity to Gal-3 on the surface of microbubbles to prepare a microbubble Gal-3-C12@lipo MBs that can target highly invasive PTC for specific diagnosis of highly invasive PTC. The diameter of the Gal-3-C12@lipo MBs was about 1.5 microns and can remain stable within 4 hours after preparation, which was conducive to stable circulation *in vivo* and reach the tumor tissue. In the targeting experiment *in vitro*, the cellular uptake of Gal-3-C12@lipo MBs was 2.8 times higher than that of non-targeted Lipo MBs, which showed superior targeting of Gal-3 positive PTC and was beneficial to the contrast-enhanced ultrasound imaging of highly invasive PTC *in vivo*. The cytotoxicity test of Gal-3-C12@lipo MBs showed that the microbubbles had good biosafety, and the modified peptides had no significant effect on cell viability, which was beneficial to clinical transformation.

To verify the targeted contrast-enhanced ultrasound imaging ability of Gal-3-C12@lipo MBs *in vivo*, a BCPAP tumor model of BCPAP was established. Firstly, the section staining of BCPAP tumor confirmed that the tumor model was indeed highly expressed Gal-3, which laid the foundation for targeted imaging of Gal-3-C12@lipo MBs. Then, the mice bearing BCPAP tumor were injected *via* tail vein with Lipo MBs and Gal-3-C12@lipo MBs, respectively, and the contrast-enhanced ultrasound imaging of tumor was monitored during the entire injection process. The Gal-3-C12@lipo MBs that entered into the tumor were not only more than Lipo MBs, but also lasted longer as demonstrated by the mean line, peak enhancement, TTP, WiPI, WiAUC, WoAUC and WiWoAUC. The results showed that Gal-3-C12@lipo MBs can significantly better target highly aggressive PTC than Lipo MBs, bringing about that the echo intensity of targeted microbubble group was significantly higher than that of non-targeted microbubble group. Since this BCPAP tumor had shown to be highly Gal-3 expressing, Gal-3-C12@lipo MBs were quite possible to identify highly invasive PTC.

However, there were also pitfalls in this study. For example, the contrast enhancement in the tumors seemed less intense than that in the background tissues. We speculated that this was because PTC was a tumor that lacks blood supply, there the blood vessels in tumor tissue were not as abundant as those in surrounding tissue. When microbubbles entered into mice through blood vessels, there were not as many microbubbles in tumor tissue as those in surrounding tissue. Therefore, as we saw, the enhancement intensity of tumor was lower than that in surrounding tissues. The data in **Table 1** were obtained from the tumor tissue compared with the surrounding tissues. The enhancement intensity of the tumor after injection of targeted contrast agent was stronger than that after injection of non-targeted contrast agent, indicating that more targeted contrast agents had entered the tumor tissues, which was consistent with the results of cell experiment. In clinical practice, we need to further add the data for differential diagnosis of benign and malignant thyroid nodules. Thus, further experiments are needed to verify it.

To sum up, we designed and prepared targeted microbubble Gal-3-C12@lipo MBs for specific contrast-enhanced ultrasound imaging diagnosis of highly invasive PTC, representing an innovative diagnostic method for *in vivo* detection and biologic characterization of thyroid nodules. In addition, the successful diagnosis of highly invasive PTC with targeted microbubbles combined with CEUS had important reference value for the stratified diagnosis of other tumors.

## Materials and methods

### Reagents and instruments

1,2-distearoyl-sn-glycero-3-phosphocholine (DSPC), 1,2-distearoyl-sn-glycero-3-phosphoethanolamine-N-[methoxy(polyethyleneglycol)-2000] (DSPE-PEG2000), 1,2-distearoyl-sn-glycero-3-phosphoethanolamine-N-[methoxy (polyethylene glycol)-2000]-fluorescein (DSPE-PEG2000-FITC), ethylene triol, and propylene glycol were purchased from Ruixi Biotechnology (Xi’an, China). Cell counting kit-8 (CCK-8) and 4,6-diamidino-2-phenylindole (DAPI) was obtained from Solarbio Inc. (Cambridge, MA, USA). Gal-3 targeted C12 peptide (ANTPCGPYTHDCPVKR) was obtained from Nanjing TG peptide Biotechnology Co., Ltd. (Nanjing, China).

A clinical ultrasound system (Siemens, Acuson Sequoia) was used to acquire all images in this study. B-mode images were collected using a 10L4 linear array transducer in spatial compounding mode as a mean for selecting the regions of interest (ROI) in each image plane. Microbubbles were imaged in cadence pulse sequencing mode, which is a nondestructive contrast-specific imaging technique available on commercial scanners. For all contrast imaging, the transducer was operating at a mechanical index of 0.08. All images were saved in Digital Imaging and Communications in Medicine (DICOM) file format.

### Mice and cells

The 5-week-old female BALB/c nude mice were purchased from Beijing Vital River Laboratory Animal Technology Co., Ltd. Each animal study was conducted in accordance with the protocols approved by the Institutional Animal Care and Use Committee at the China-Japan Friendship Hospital (No. Zryhyy12-20-01-9). The human BCPAP cell line was purchased from the BNCC company (Beijing, China). The cells were cultured in complete RPMI-1640 medium (Gibco, Thermo Fisher Scientific, USA) containing 10% fetal bovine serum (FBS, Gibco) and 1% penicillin-streptomycin (Gibco) in a cell incubator (Thermo, USA) with 5% CO_2_ and 95% air at 37°C.

### Synthesis of Lipo MBs and Gal-3-C12@lipo MBs

Lipo MBs was prepared through thin-film hydration method followed by mechanical shaking (28). 0.72 mg DSPC and 0.28 mg DSPE-PEG2000 (with molar ratio of about 9:1) were firstly dissolved in 0.25 mL CHCl_3_, followed by evaporation into film. Then, the film was dispersed into 1.2 mL PBS (pH 7.4) under water bath sonication to form lipid nanoparticles. The formed lipid nanoparticles were subsequently transferred into a glass vial for filling with SF_6_ gas and sealing. After mechanically shaking *via* an agitator, Lipo MBs could be obtained.

Gal-3-C12@lipo MBs were prepared in the similar ways as above. Briefly, 0.6 mg DSPC and 0.2 mg DSPE-PEG2000-NHS (with molar ratio of about 9:1) were used to prepare the lipid nanoparticles, followed by incubation with 0.2 mg targeted peptide C12 for 1 hour at 30°C (molar ratio of DSPE-PEG2000-NHS/C12 peptide =1:2). Then, the formed targeted C12-lipid nanoparticles were dialyzed to remove unconnected peptide and used to prepare Gal-3-C12@lipo MBs by the same way of inflating and shaking (29).

### Characterization of Gal-3-C12@lipo MBs

The size and concentration of Gal-3-C12@lipo MBs were measured through an AccuSizer (Particle Sizing Systems Inc., USA). The morphologies of Gal-3-C12@lipo MBs were observed by a fluorescence microscope (Nikon, Japan) (30).

To evaluate the stability of Gal-3-C12@lipo MBs, the size and concentration of Gal-3-C12@lipo MBs were monitored at 0, 1, 2, and 4 h after preparation.

### *In vitro* biocompatibility

For demonstrating the biocompatibility of Gal-3-C12@lipo MBs, BCPAP cells were seeded in 96-well plates (8×10^3^ cells/well) and incubated overnight. Then, the original medium was discarded and replaced with culture medium containing Gal-3-C12@lipo MBs at different concentrations of 0, 12.5, 25, 50, 100, 200 and 300 μg mL^-1^, followed by another 24 hours incubation. Subsequently, cytotoxicity was evaluated by CCK-8 assay.

### Evaluation of *in vitro* targeting specificity

Gal-3-C12@lipo MBs were labelled with FITC by self-assembly with 1% DSPE-PEG2000-FITC to evaluate the targeting ability and FITC labelled Lipo MBs were used as control. The BCPAP cells were seeded (8×10^5^ cells/well) in 20 mm glass bottom cell culture dishes (Nest Biotechnology, Wuxi, China) and incubated for 24 h. Then, the medium was replaced with fresh medium containing 100 μg mL^-1 FITC^Gal-3-C12@lipo MBs or ^FITC^Lipo MBs and incubated for another 6 hours. Afterwards, the cells were washed with PBS and stained with DAPI for 10 min. CLSM (Zeiss LSM 900) was applied to observe the fluorescence pictures of cells.

To quantitatively study the targeting specificity of Gal-3-C12@lipo MBs, BCPAP cells were seeded in 6-well dished (1×10^6^ cells/well) and incubated for 12 hours. Subsequently, fresh medium containing 100 μg mL^-1 FITC^Gal-3-C12@lipo MBs was added to the cells. After 6 hours incubation, the cells were washed with PBS and digested with trypsin to collect cells for further flow cytometry. ^FITC^Lipo MBs were used as control.

### Tumor model establishment

The BCPAP subcutaneous tumor models were established by injecting 5×10^6^ BCPAP cells/per mouse on the right flanks of the BALB/c nude mice. The experiments were carried out until the tumor volume achieving 75~100 mm^3^.

### *In vivo* CEUS

BALB/c nude mice bearing BCPAP subcutaneous tumor were prepared for imaging by anesthetization with 2% inhaled isoflurane mixed with oxygen. The tumor-forming nude mice were randomly divided into two groups: Lipo MBs group (n=6) and Gal-3-C12@lipo MBs group (n=6). The nude mice in Lipo MBs group were injected with Lipo MBs and those in Gal-3-C12@lipo MBs group were injected with Gal-3-C12@lipo MBs. A 24-gauge catheter was inserted into the tail vein for the purpose of administering microbubbles into the circulatory system of the animal. For each injection, 100 μL Gal-3-C12@lipo MBs or Lipo MBs (800 μg mL^-1^) were administered and flushed with 100 μL of sterile saline. The entire CEUS procedure was recorded for each tumor from the start of the injection until the contrast agent couldn’t be observed.

### Image analysis

The CEUS studies were saved on the hard disc in the ultrasound system (native data) and transferred to a computer for further quantitative analyses with advanced ultrasound quantification software (VueBox^®^; Bracco, Suisse SA, Geneva, Switzerland). We linearized DICOM cine loops, applied curve-fitting models, then evaluated time-intensity curves for the following parameters: peak enhancement (arbitrary units, AU), defined as the maximum intensity of the curve; wash-in area under the curve (WiAUC, AU); wash-out area under the curve (WoAUC, AU); wash-in and wash-out areas under the curve (WiWoAUC, AU); wash-in rate (WiR), in terms of maximum slope (AU); wash-out rate (WoR), in terms of minimum slope (AU); rise time (s); fall time (s); mean transit time local (mTTl, s); time to peak (TTP, s); wash-in perfusion index (WiPI), defined as WiAUC/rise time; and quality of fit (%) between echo-power signal and f (t). Data for these variables were plotted as parametric images (color-coded maps), to which time-intensity curves were fitted and linearized.

Image analyses were performed quantitatively and qualitatively by two radiologists, one with more than 5 years and the other with more than 10 years of experience in ultrasound imaging. The two radiologists placed the ROIs independently, and each drew two ROIs on the contrast enhanced images with reference to the grayscale ultrasound image. ROI 1 included the entire tumor boundary on gray-scale ultrasound, while avoiding the surrounding parenchyma. ROI 2 encircled the normal-appearing parenchyma including the same image acquisition plane as far from the tumor as possible from normal parenchyma. Analysis was performed in dual-screen mode for B-mode and contrast-enhanced ultrasonography, starting with synchronization of B-mode and contrast-enhanced imaging of the first arterial loop. Contrast-enhanced ultrasound features were assessed with the assistance of color-coded imaging in VueBox. The largest value of peak enhancement was colored dark red, and the lowest value was colored dark blue. The lowest values of rise time and TTP were colored dark red, and the largest values were colored dark blue.

### Histology and immunohistochemistry staining of tumors

Hematoxylin and eosin (H&E) staining was performed for histological evaluation of BCPAP cancer. The cryosections were rinsed with distilled water and stained with hematoxylin, followed by rinsing with running tap water. After differentiation in 0.3% acid alcohol and another rinse, the sections were stained with eosin for 2 minutes. Finally, the sections were consecutively dehydrated, cleared and mounted, and were examined under an optical microscope (Leica, DMI3000).

Immunohistochemical staining was conducted for evaluating the expression of Gal-3 of BCPAP tumors. Briefly, 4-µm consecutive tissue sections were obtained from tumors excised from each animal used for CEUS imaging studies. Antigen retrieval microwave treatment of tissue sections in 0.01 mol/L citrate buffer pH 6.0 was applied for three cycles of 3 to 5 minutes each at 750 W. A purified horseradish peroxidase conjugated mAb to Gal-3 was used at concentration range of 5~10 mg mL^-1^ in direct immunoperoxidase. The enzymatic activity was visualized with 3, 30-diamino-benzidine.

### Statistical analysis

Quantitative data represent the mean ± SD. SPSS software (V.22.0) was used to analyze the data, and GraphPad Prism V.8.0 (La Jolla, California, USA) was used to create the graphs. A Student’s t-test or a Mann-Whitney U test was used to evaluate statistical significance, with *p*<0.1 considered statistically significant (no significance; *p<0.1; ‡*p*<0.01; §*p*<0.001).

## Data availability statement

The raw data supporting the conclusions of this article will be made available by the authors, without undue reservation.

## Ethics statement

The animal study was reviewed and approved by the protocols approved by the Institutional Animal Care and Use Committee at the China-Japan Friendship Hospital (No. Zryhyy12-20-01-9).

## Author contributions

JM, XL and BZ contributed the conception and the design of experiments. YW, LKW and LPW performed the research. XX reviewed and edited the manuscript. All authors analyzed and interpreted the data. JM and YW wrote the paper. BZ and XL approved of the version to be published. All authors contributed to the article and approved the submitted version.
